# Effects of method and duration of restraint on stress hormones and meat quality in broiler chickens with different body weights

**DOI:** 10.5713/ajas.18.0354

**Published:** 2018-10-26

**Authors:** Siti Nadirah Ismail, Elmutaz Atta Awad, Idrus Zulkifli, Yong Meng Goh, Awis Qurni Sazili

**Affiliations:** 1 Halal Products Research Institute, Universiti Putra Malaysia, 43400 UPM Serdang, Selangor, Malaysia; 2 Institute of Tropical Agriculture and Food Security, Universiti Putra Malaysia, 43400 UPM Serdang, Selangor, Malaysia; 3 Department of Poultry Production, University of Khartoum, 13314 Khartoum North, Sudan; 4 Department of Animal Science, Universiti Putra Malaysia, 43400 UPM Serdang, Selangor, Malaysia; 5 Department of Veterinary Preclinical Sciences, Faculty of Veterinary Medicine, Universiti Putra Malaysia, 43400 UPM Serdang, Selangor, Malaysia

**Keywords:** Broiler Chickens, Restraint Method, Duration of Restraint, Body Weight, Stress, Meat Quality

## Abstract

**Objective:**

The study was designed to investigate the effects of restraint method, restraint duration, and body weight on stress-linked hormones (corticosterone, adrenaline, and noradrenaline), blood biochemical (namely glucose and lactate), and the meat quality in broiler chickens.

**Methods:**

A total of 120 male broiler chickens (Cobb 500) were assigned to a 2×3×2 factorial arrangement in a completely randomized design using two restraint methods (shackle and cone), three durations of restraint (10, 30, and 60 s), and two categories of live body weight (1.8±0.1 kg as lightweight and 2.8±0.1 kg as heavyweight).

**Results:**

Irrespective of the duration of restraint and body weight, the coned chickens were found to have lower plasma corticosterone (p<0.01), lactate (p<0.001), lower meat drip loss (p<0.01), cooking loss (p<0.05), and higher blood loss (p<0.05) compared with their shackled counterparts. The duration of restraint had significant effects on the meat initial pH (p<0.05), ultimate pH (p<0.05), and yellowness (p<0.01). The lightweight broilers exhibited higher (p< 0.001) blood loss and lower (p<0.05) cooking loss compared to the heavyweight broilers, regardless of the restraint method used and the duration of restraint. However, the interaction between the restraint method, duration of restraint, and body weight contributed to differences in pre-slaughter stress and meat quality. Therefore, the interaction between the restraint method and the duration of restraint affected the meat shear force, lightness (L*) and redness (a*).

**Conclusion:**

The duration of restraint and body weight undoubtedly affect stress responses and meat quality of broiler chickens. Regardless of the duration of restraint and body weight, the cone restraint resulted in notably lower stress, lower meat water loss, and higher blood loss compared to shackling. Overall, the findings of this study showed that restraint method, duration of restraint, and body weight may affect the stress response and meat quality parameters in broilers and should be considered independently or interactively in future studies.

## INTRODUCTION

Environmental conditions shortly before slaughter have been known to be stressful for broiler chickens and may profoundly affect the quality of their meat [1]. For example, the restraint method and slaughter weight were reported to have noticeable effects on pre-slaughter and post-slaughter physiological responses in broiler chickens [2,3]. Also, the shackling of live broiler chickens is known to be detrimental to their welfare [4]. Furthermore, it has also been reported that as the shackling duration increases, the rate of postmortem muscle glycolysis [1] and circulating corticosterone [5] increases. The heavier or older chickens are more prone to stress [3], have lower values of meat lightness (L*), lower meat ultimate pH (pH_u_), and lower shear force values [6]. During the past few years, cone restraining has been discovered to limit the movement of birds either pre-slaughter, during slaughter, or early post-slaughter. In an earlier study, cone restraint was suggested during stunning and exsanguinations to limit convulsions compared to shackling [7]. Moreover, it has been found that placing birds in a cone restraint followed by stunning successfully limited the struggling of chickens [7] and minimized the incidence of muscle hemorrhage [2]. However, no previous study has investigated the effects of cone restraining without stunning on the physiological stress response or meat quality in broiler chickens. Therefore, this study was conducted to determine the effects of pre-slaughter restraint method, duration of restraint, and body weight during slaughter on stress-linked hormones, blood biochemical, and the meat quality in broiler chickens. It was hypothesized that restrain method, duration of restraint, and body weight would generate changes in the physiological stress responses and meat quality of broilers.

## MATERIALS AND METHODS

### Birds, management, and experimental design

This study was conducted in accordance with the guidelines developed by the Research Policy on Animal Ethics of Universiti Putra Malaysia. One-day-old male broiler chicks (Cobb 500) were supplied from a commercial hatchery and randomly assigned to groups of 20 in battery cages with wire floors for the first two weeks, reduced to 10 birds per cage for the rest of the experiment. Each cage measured 85×100×50 cm (width× length×height) and was equipped with one feeder and one drinker. All chicks received the same standard broiler starter (day 1 to 21) and finisher (day 22 to 42) diets. The feed was supplied from a local feed mill (Gold Coin Feedmills (M) Pty. Ltd., North Port, Port Klang, Selangor, Malaysia). Throughout the rearing period, the birds were provided with *ad libitum* amount of feeds and drinking water, and the lighting was continuous. A total of 120 birds were used in this study, which were assigned to a 2×2×3 factorial arrangement in an entirely randomized design with two levels of restraint method (shackle and cone), three levels of restraint duration (10, 30, and 60 s), and two levels of body weight at slaughter (1.8±0.1 kg, as lightweight and 2.8±0.1 kg, as heavyweight). There were 10 birds per each method-duration-weight subgroup. To obtain the two categories of body weight, the birds were slaughtered at two different ages (day 35 for the lightweight group and day 42 for the heavyweight group). Accordingly, on day 35, a total of 60 birds of about the same size were randomly selected to serve as the lightweight group, and transported to the university slaughterhouse (Department of Animal Science, Faculty of Agriculture, Universiti Putra Malaysia) for restraining and slaughtering. Next, on day 42, another 60 birds of about the same size were randomly selected to serve as the heavyweight group, and transported to the slaughterhouse for restraining and slaughtering. Both groups were slaughtered at the same time under the same duration to eliminate the effects of daylight.

### Restraining, slaughtering, and sampling

On days 35 (lightweight birds) and 42 (heavyweight birds) and following 10 min of transportation of the birds and 60 min of lairage, the birds were weighed individually, with their weights recorded as the live body weight. The chickens were then subjected to restraining, either by the cone or shackle method for different durations (10, 30, or 60 s), before being slaughtered. Exsanguination blood samples were collected into commercial tubes containing ethylenediaminetetraacetic acid as an anticoagulant and kept temporarily in an ice bucket. The blood samples were then centrifuged at 3,000×*g* at 4°C for 15 min, and the recovered plasma fraction was stored at −80°C until subsequent analysis. After exsanguination for 90 s, the dead birds were then individually weighed, with their weights recorded as the weight after slaughter.

### Blood biochemical and hormones

The physiological stress responses were determined through biochemical parameters (glucose and lactate) as well as plasma hormones (corticosterone, adrenaline, and noradrenaline). Glucose and lactate levels were determined using an automatic analyzer (Automatic Analyzer 902, Hitachi, Feldkirchen, Germany) and the corticosterone concentrations were measured using a Corticosterone HS (high sensitivity) EIA (Immunodiagnostic System Limited, Boldon Colliery, UK) kit, following the manufacturer’s protocol. The adrenaline concentrations were measured using an Adrenaline Plasma ELISA High Sensitive kit # BA E-4100 (LDN, Nordhorn, Germany) while the noradrenaline concentrations were measured using a Noradrenaline Plasma ELISA High Sensitive kit # BA E-4200 (LDN, Germany), following the manufacturer’s protocols and instructions.

### Blood loss

During the 90 s of exsanguination, the blood loss of individual birds was determined by the difference in their body weight before and after slaughtering [8]. The percentages of blood loss were calculated using the following equation:

Blood loss (%)=[(W1-W2)/W1]×100

Where,

W1 = live weight and W2 = weight after slaughter.

### Muscle pH

Samples for the determination of pH were taken from the right *pectoralis major* at 45 min (initial pH) and at 24 h (ultimate pH) post-mortem. The pH value was measured using a modified method [9]. Next, 0.5 g of cone group meat samples were homogenized in 10 mL of 4°C distilled water, and the pH was measured using a pH meter equipped with an electrode (Mettler Toledo, Columbus, OH, USA).

### Color

Next, meat for color analysis was sampled from the right pectoralis major muscle at 24 h post-mortem and objectively measured using ColorFlex (HunterLab, Reston, VA, USA). The samples were then exposed for 30 min at ambient room temperature (24°C) to allow blooming before being trimmed to a thickness of 1.5 cm. The triplicate color coordinate values of L* (lightness), a* (redness), and b* (yellowness) were then measured on the cut surface of the muscle samples and recorded [10].

### Water holding capacity

#### Drip loss

For drip loss, samples were taken from the left *pectoralis major* at 7 min post-mortem and determined [11]. Next, the meat samples were weighed and recorded as W1 (initial weight) before being placed into a vacuumed polyethylene bag, sealed and stored at 4°C for 24 h. The samples were then taken out of the plastic bag and the surface of the sample was gently blot dried using soft tissue paper before being reweighed and recorded as W2. To determine the drop loss percentage of the 24 h post chilled muscle, the following equation was applied [11]:

Drip loss (%)=[(W1-W2)/W1]×100

Where,

W1 = initial weight and W2 = final weight.

#### Cooking loss

Samples to determine drip loss were taken from the left *pectoralis major* at 24 h post-mortem. Samples were weighed and recorded as W1 (initial weight). After the determination of the drip loss percentage, the samples were placed into polyethylene bags and cooked in a water bath pre-set at 80°C for 30 min. The cooked samples were then removed from the labeled polyethylene bags, pre-cooled, re-weighed and recorded as W2 (final weight). The cooking loss percentages were then calculated and determined based on the difference between W1 and W2 using the following equation [11]:

Cooking loss (%)=[(W1-W2)/W1]×100

Where,

W1 = initial weight and W2 = final weight.

### Texture

Samples from the cooking loss determination were used to measure the texture of the sample via shear force analysis. Each sample was cut into three sub-samples for the measurement having a similar thickness. Accordingly, the sample was cut based on the shape and fiber orientation [12]. Each sample was sheared once perpendicularly to the fibers at a speed of 1.0 mm/s with a Volodkevitch bite jaw attached to a TA.HDplus texture analyzer (Stable Micro System, Surrey, UK) fitted with a 5 kg load cell.

### Statistical analysis

The data were subjected to analysis of variance using the general linear model procedure in SAS [13] and analyzed using the restraint method, duration of restraint, the bird’s body weight, and their interactions as the main effects. The differences between group means were analyzed applying Tukey’s test where the statistical significance is considered at p≤0.05.

## RESULTS

### Blood biochemical

Plasma concentrations of glucose and lactate as affected by the restraint method, duration of restraint, and body weight are presented in Table 1. Main effects of restraint method, duration of restraint, and body weight, and all possible interactions were not significant (p>0.05) for glucose. The results on plasma lactate, however, showed a significant effect of restraint method (p<0.001) as plasma lactate in the shackle restraint group was higher compared to the cone restraint group. Chickens restrained for 10 s had significantly (p<0.001) higher plasma lactate compared to those restrained for 30 and 60 s. Regardless of the restraint method and the duration of restraint, plasma lactate was significantly (p<0.001) higher in lightweight chickens compared to heavyweight chickens. Further, significant (p< 0.05) 2-way interactions between the duration of restraint and body weight were observed for plasma lactate.

### Stress-linked hormones

The effects of restraint method, duration of restraint and body weight on plasma concentrations of corticosterone, adrenaline, and noradrenaline are shown in Table 1. Main effects of body weight, duration of restraint, and all possible interactions were not significant (p>0.05) for corticosterone. However, the restraint method significantly (p<0.01) affected the corticosterone level as the shackled birds had a higher corticosterone concentration than their coned counterparts. Main effects showed no significant (p>0.05) effect on the adrenaline or noradrenaline concentrations. Significant (p<0.05) 2-way interactions between the restraint method and the duration of restraint were observed for noradrenaline.

### Blood loss

Regardless of the duration of restraint and body weight, the restraint method significantly (p<0.05) affected blood loss as restraining the birds using the cone restraint resulted in higher blood loss as compared to restraining the birds using a shackle restraint (Table 2). Furthermore, the duration of restraint did not affect (p>0.05) the blood loss. However, regardless of which restraint method was used and the duration of restraint, the body weight of the birds before slaughter significantly (p<0.001) affected the animal’s blood loss. The highest blood loss was found in the lightweight group of birds as compared to the heavyweight group (Table 2).

### Muscle pH values

Table 2 shows the differences in the pH of the right *pectoralis major* muscles obtained from the animals subjected to the shackle and cone restraint methods after 45 min and 24 h post-mortem. The restraint method and body weight did not affect (p>0.05) the initial muscle pH (45 min post slaughter). However, a significant effect (p<0.01) of the duration of restraint was observed among those chickens that were shackled but not observed in the coned chickens (Table 2). Accordingly, the highest initial muscle pH was recorded in the samples from the chickens that were restrained for 60 s.

Unlike the restraint method, both the body weight and duration of restraint significantly (p<0.001 and p<0.05, respectively) affected the ultimate pH of the breast muscle. The pH_u_ was found to be significantly higher in the heavy group of birds compared to the light group. Also, the pH_u_ was noted to be higher in the birds that were restrained for 60 s as compared to those birds that were restrained for 30 s (Table 2). There was significant (p<0.05) interaction between the duration of restraint and body weight on the muscle ultimate pH.

### Meat quality measurements

#### Drip loss

At day one post-mortem, the duration of restraint and slaughter weight did not significantly affect the drip loss. However, the restraint method significantly (p<0.05) influenced the drip loss where the muscle samples from the shackle group indicated a higher drip loss percentage compared to their counterparts from the cone group (Table 3).

#### Cooking loss

At day one post-mortem, the cooking loss was found to be affected by both the slaughter weight and restraint method, but not the duration. The cooking loss was significantly (p<0.05) higher in the heavyweight group as compared to the lightweight group. Also, the shackle group had the higher (p<0.05) cooking loss than those birds in the cone group (Table 3).

#### Shear force values

All possible 2-way interactions were significant for shear force (Table 3). The main effect of body weight had no significant effect (p>0.05) on shear force. However, both the restraint method and duration of restraint significantly (p<0.001) affected the shear force values. Shear force was higher in the shackled chickens compared to the coned chickens. Regardless of the restraint method and the body weight, the shear force value of the chickens restrained for 10 s was higher compared to their counterparts restrained for 30 and 60 s.

#### Color values

In this study, the parameter for color consists of lightness (L*), redness (a*), and yellowness (b*). Main effects of restraint method (p<0.001), duration of restraint (p<0.001), body weight (p<0.001), the 2-way interactions between restraint method and the duration of restraint (p<0.01), and the 2-way interactions between the duration of restraint and body weight (p<0.05), were significant for L* (Table 3). The lightness was significantly higher in shackle restrained chickens compared to cone restrained chickens, lightweight chickens compared to heavyweight chickens, and 10 s restrained chickens compared to 30 and 60 s restrained chickens.

The redness of the chicken meat was also found to be in fluenced by the main effects of restraint method (p<0.01), duration of restraint (p<0.001), body weight (p<0.001), the 2-way interactions interaction between the restraint method and the duration of restraint, and the 3-way interactions (p< 0.01) (Table 3). The redness value was higher in coned chickens compared to shackled chickens, lightweight chickens compared to heavyweight chickens, and 30 s and 60 s restrained chickens compared to10 s restrained chickens.

Main effects of restraint method (p <0.001), duration of restraint (p<0.05), body weight (p<0.001), and the 2-way interactions interaction between the restraint method and the body weight, were significant for the yellowness of the chicken meat (Table 3). The yellowness value was significantly higher in coned chickens compared to shackled chickens, lightweight chickens compared to heavyweight chickens, and 60 s restrained chickens compared to 10 s restrained chickens.

## DISCUSSION

Glucose is an essential metabolic substrate that is primarily produced by the breakdown of glycogen in the liver. As the broiler chickens undergo stress, the glucose metabolism will be promoted and coupled with changes in muscle glycogen, lactic acid, and adenosine triphosphate enzyme contents [14]. It was reported that chickens with heavier slaughter weights contained higher plasma glucose than lighter chickens [3]. Therefore, this suggests that the heavier the chicken, the more prone they are to pre-slaughter stress. The present study, however, found no significant effect of slaughter weight, restraint method, or duration of restraint on the plasma glucose concentration, although the lactate concentration had been found to be affected by all three factors in the present study. Furthermore, it was observed that shackling resulted in higher levels of plasma lactate than by cone restraining. Indeed, this could be due to struggling, or wing flapping (an escape or discomfort behavior) that occurred during shackling restraint. The body restraint method using a sorting board has reportedly reduced blood lactate in pigs [15]. Interestingly, higher plasma glucose concentrations were observed in broiler chickens slaughtered at >2.4 kg than from those slaughtered at <2.0 kg [3].

The act of shackling may induce stress in poultry and it is recognized that exposure to stress can trigger the neuroendocrine system of vertebrate animals [16]. Accordingly, stress involves an immediate (0 to 20 min) response by catecholamine neurotransmitters (e.g., noradrenaline) and a medium-term (3 to 120 min) response by non-genomic and subsequently the genomic effects of corticosteroid hormones (e.g., corticosterone in birds) [17]. Also, at the early stage of the stress response, both catecholamines and corticosteroids are elevated, while at a later stage of the stress response, only the corticosteroids remain elevated [18]. This explains why in the present study, only corticosterone was affected using the shackling method and not adrenaline and noradrenaline. However, it is possible that the birds were, in fact, exposed to stress long before shackling and cone restraining were performed, most likely during transportation from the farm to the slaughterhouse.

A poor bleed-out in broiler chickens can also be observed by hemorrhagic conditions of the meat [8]. Apparently, hemorrhages found in broiler carcasses can be caused by the occurrence of blood circulation disturbances in the broiler chicken [8]. Moreover, higher breast muscle hemorrhage scores were observed in Hybro broilers (heavier) compared to Ross broilers (lighter) [8]. Further, the authors in [2] documented that birds shackled in a moving line for 10 s lost more blood than those birds restrained in a cone for the same duration, regardless of the type of stunning method employed. Also, hanging could facilitate rapid bleeding through severe struggling (straightening up and wing flapping) by birds in a shackle line [19]. However, it is understood that wing flapping only occurs during the first few seconds after shackling, while some birds tend to struggle within just a few seconds of shackling. Notwithstanding, many birds subsequently resume wing flapping if they are suddenly exposed to sunlight, sudden jolting or experiencing electric shocks at the water bath [19]. The reaction of hanging has also been shown to be intensified by environmental factors such as rough hanging, unsuitable shackles or separation from familiar counterparts [19].

It was reported that broilers slaughtered at 32 days (younger) had significantly lower pH values at 24 h postmortem than those birds slaughtered at 42 days (older) [6]. Lower muscle pH at 4 h post-mortem was observed in birds slaughtered at 42 days compared with those birds slaughtered at 53 days [20]. The differences in ultimate pH values could be related to changes in the post-mortem muscle metabolism which in turn, could have resulted in differences in the rate of pH decline. Further, it appears that a higher rate of decrease in pH may also lead to lower ultimate pH in broilers [21], thereby suggesting that the decline in pH of the breast muscle tends to decrease as the bird’s increases in age. Further, lower initial pH was documented in shackled birds compared to those restrained by the cone which could be due because of struggling during shackling [2]. The previous study showed that the initial rate of pH could be accelerated by free wing flapping [22]. Notably, struggling and wing flapping increases the lactate concentration in breast muscle, thus lowering the muscle pH at 15 min post-mortem [23].

Color is a significant factor that influences the consumer’s acceptability of meat and is a useful tool for assessing meat at the time of purchase. It was reported that meat redness of broilers decreased with age [24]. Furthermore, it was documented that breast muscle hypertrophy likewise occurs as birds age [25], causing myofibers to be enlarged which reduces the density of the capillary peripheral to the myofibers [26]. This could contribute to the reduced redness of meat. The color of breast meat has often been related to the post-mortem kinetics of muscle pH decline. The lightest breast meat is characterized by the least decline in pH [25] while broiler breast muscles with lower pH appear less red than those with higher pH [27]. As a primary source of nitrogen species, nitric oxide (NO) is a signaling molecule which plays multiple biological functions. Traditionally, both nitrates and nitrites have been used for the curing of meat for increasing meat’s shelf-life and improving its color by decreasing the yield of NO. The effects of NO on the quality of broiler chickens meat during storage were investigated by treating the meat with NO synthase inhibitor or NO enhancer [28]. The authors concluded that NO could play a primary role in modulating the quality of fresh broiler breast meat during refrigerated storage. In fresh meat, the level of endogenous NO was postulated to influence the quality of meat including the color with pre-slaughter handling to play a profound role in the NO level in muscle cells [29]. Thus, in this study, changes in color coordinates could be also associated with the level of NO despite the inability to specify the exact mechanism (s) by which NO could affect meat quality [29].

It was reported that shear force of lighter birds slaughtered at 32 days was significantly higher than the shear force of heavier birds slaughtered at 42 days and that the muscle fiber cross-sectional area increases with age [6]. The cross-sectional area of these giant fibers is usually three to five times larger than the normal fibers found in aged birds [30]. The smaller fiber diameters in younger birds may allow higher packing density and thereby increasing the toughness of their muscles [6]. Wing restraint treatment stretches the breast muscles and prevents contraction, resulting in longer sarcomeres thus improving the tenderness of the meat [31]. Also, birds struggling at slaughter can significantly decrease the water-holding capacity of broiler chicken [32]. However, struggling during shackling is not necessarily caused by the duration of shackling. Many causes of wing flapping during shackling were proposed including the tight fitting shackles on the birds’ shanks, electric shocks at the water bath stunner before stunning, sudden bright sunlight, bends in the line, temporary loss of visual contact between neighbouring birds, and unevenness in the line which jolted the birds [19].

## CONCLUSION

The results of this study demonstrate that the restraint method, duration of restraint and body weight affect stress responses and the meat quality of broiler chicken. The restraint method was found to significantly affect blood loss, plasma lactate and corticosterone concentrations, meat drip loss and cooking loss. Compared to cone restraining, the shackling of birds results in lower blood loss, higher plasma lactate and corticosterone levels, higher meat drip loss and higher cooking loss. Furthermore, body weight at slaughter affects blood loss and meat cooking loss. It was also found that lighter birds lost more blood compared to heavier birds. The interaction between the restraint method and the duration of restraint was also found to affect the meat shear force, lightness (L*) and redness (a*). The broilers that were restrained in the cone for over 30 s had lower drip loss and shear force, darker and redder meat compared to the shackled broilers at 30 s. The interaction between the restraint method and body weight was found only to affect meat yellowness (b*). The meat from birds restrained in the cone had higher yellowness than the meat from the birds restrained using the shackle method in both body weight groups. The interaction between the duration of restraint and body weight also affected the plasma lactate concentration and meat lightness. The lighter birds restrained for 10 s and 60 s had higher blood lactate and were lighter in color than the heavier birds restrained for 60 s, while the interaction between the restraint method, duration of restraint and body weight was found to affect meat redness. The highest redness value was observed in the lighter birds restrained by the cone for 60 s.

## Figures and Tables

**Table 1:** Plasma metabolites and stress-linked hormones as affected by restraint method (R), duration of restraint (D), and body weight (W) prior slaughter in broiler chickens

Restraint method	Restraint duration (s)	Body weight1)	n	Glucose (mmol/L)	Lactate (mmol/L)	Corticosterone (ng/mL)	Adrenaline (ng/mL)	Noradrenaline (ng/mL)
Interaction effects								
Cone	10	Light	10	13.85	5.55	1.90	2.40	3.06
Cone	10	Heavy	10	13.74	4.00	1.85	2.31	3.18
Cone	30	Light	10	13.98	2.84	1.90	2.39	3.27
Cone	30	Heavy	10	13.85	2.45	1.87	2.14	3.15
Cone	60	Light	10	14.21	3.58	1.92	1.95	3.11
Cone	60	Heavy	10	14.56	2.04	1.92	2.27	3.10
Shackle	10	Light	10	15.50	6.80	1.95	2.40	3.21
Shackle	10	Heavy	10	14.26	4.96	1.93	2.02	3.17
Shackle	30	Light	10	14.29	4.10	1.93	2.35	2.96
Shackle	30	Heavy	10	13.62	3.84	1.97	2.19	3.18
Shackle	60	Light	10	13.61	5.98	1.97	2.35	3.30
Shackle	60	Heavy	10	14.17	3.02	1.90	2.34	3.20
SEM2)				0.60	0.46	0.02	0.13	0.08
Main effects								
Cone			60	14.03	3.41^b^	1.89^b^	2.24	3.15
Shackle			60	14.24	4.78^a^	1.94^a^	2.28	3.17
SEM3)				0.24	0.19	0.01	0.05	0.03
	10		40	14.34	5.33^a^	1.91	2.28	3.16
	30		40	13.94	3.31^b^	1.92	2.27	3.14
	60		40	14.14	3.66^b^	1.93	2.23	3.17
	SEM4)			0.29	0.23	0.01	0.07	0.04
		Light	60	14.24	4.81^a^	1.93	2.31	3.15
		Heavy	60	14.03	3.39^b^	1.91	2.21	3.16
		SEM5)		0.24	0.19	0.01	0.05	0.03
ANOVA (p-value)								
R				0.54	***	**	0.66	0.60
D				0.63	***	0.34	0.81	0.79
W				0.55	***	0.08	0.21	0.84
R×D				0.17	0.67	0.15	0.12	*
R×W				0.48	0.33	0.61	0.23	0.75
D×W				0.38	*	0.53	0.07	0.57
R×D×W				0.73	0.48	0.06	0.47	0.06

SEM, standard error of the mean; ANOVA, analysis of variance.

1) Light (1.8±0.1 kg), heavy (2.8±0.1 kg).

2) Restraint method, restraint duration, and body weight effect (n = 10).

3) Restraint method effect (n = 60).

4) Restraint duration effect (n = 40).

5) Body weight effect (n = 60).

* p<0.05;

** p<0.01;

*** p<0.001.

a–d Means within a column-subgroup with no common superscripts are significantly different at p<0.05.

**Table 2:** Blood loss, breast muscle initial (pH_45_) and ultimate pH (pH_u_) as affected by restraint method (R), duration of restraint (D), and body weight (W) prior slaughter in broiler chickens

Restraint method	Restraint duration (s)	Body weight1)	n	Blood loss (%)	pH_45_	pH_u_
Interaction effects						
Cone	10	Light	10	3.09abc	6.16^ab^	5.87
Cone	10	Heavy	10	3.09abc	6.08^ab^	5.87
Cone	30	Light	10	3.81^a^	6.08^ab^	5.84
Cone	30	Heavy	10	3.07abc	6.07^ab^	6.04
Cone	60	Light	10	3.68^ab^	6.08^ab^	5.87
Cone	60	Heavy	10	2.80^bc^	6.13^ab^	6.07
Shackle	10	Light	10	3.52abc	6.02^b^	5.87
Shackle	10	Heavy	10	2.77^bc^	6.09^ab^	5.92
Shackle	30	Light	10	2.74^bc^	6.06^ab^	5.86
Shackle	30	Heavy	10	2.70^c^	6.04^b^	5.95
Shackle	60	Light	10	3.46abc	6.23^a^	5.89
Shackle	60	Heavy	10	2.97abc	6.12^ab^	6.02
SEM2)				0.20	0.04	0.04
Main effects						
Cone			60	3.26^a^	6.10	5.93
Shackle			60	3.03^b^	6.09	5.92
SEM3)				0.09	0.02	0.02
	10		40	3.12	6.08^ab^	5.89^b^
	30		40	3.08	6.06^b^	5.92^ab^
	60		40	3.23	6.14^a^	5.96^a^
	SEM4)			0.11	0.02	0.02
		Light	60	3.38^a^	6.10	5.87^b^
		Heavy	60	2.90^b^	6.09	5.98^a^
		SEM5)		0.09	0.02	0.02
ANOVA (p-value)						
R				*	0.80	0.69
D				0.57	*	*
W				***	0.49	***
R×D				*	*	0.57
R×W				0.62	0.89	0.34
D×W				0.47	0.82	*
R×D×W				*	*	0.30

SEM, standard error of the mean; ANOVA, analysis of variance.

1) Light (1.8±0.1 kg), heavy (2.8±0.1 kg).

2) Restraint method, restraint duration, and body weight effect (n = 10).

3) Restraint method effect (n = 60).

4) Restraint duration effect (n = 40).

5) Body weight effect (n = 60).

* p<0.05;

*** p<0.001.

a–d Means within a column-subgroup with no common superscripts are significantly different at p<0.05.

**Table 3:** Meat quality parameters as affected by restraint method (R), duration of restraint (D), and body weight (W) prior slaughter in broiler chickens

Restraint method	Restraint duration (s)	Body weight1)	n	Drip loss (%)	Cooking loss (%)	Shear force (kg)	L*	a*	b*
Interaction effects									
Cone	10	Light	10	2.11	20.26	1.20	50.62	5.57^cd^^e^	20.59
Cone	10	Heavy	10	1.63	27.90	1.26	48.85	3.69^f^	16.28
Cone	30	Light	10	1.27	19.30	1.13	48.70	7.49^b^	22.71
Cone	30	Heavy	10	1.43	20.33	1.16	43.67	4.47^ef^	17.33
Cone	60	Light	10	1.14	19.33	0.98	46.67	9.02^a^	22.41
Cone	60	Heavy	10	1.37	19.77	1.14	42.02	5.17^d^^ef^	17.31
Shackle	10	Light	10	1.79	22.18	1.38	51.07	6.97^bc^	17.38
Shackle	10	Heavy	10	1.35	25.66	1.22	50.06	3.87^f^	15.54
Shackle	30	Light	10	2.01	22.06	1.19	51.03	6.13bcd	19.44
Shackle	30	Heavy	10	2.20	23.01	1.13	47.34	4.47^ef^	14.60
Shackle	60	Light	10	2.34	22.34	1.19	50.31	6.50bcd	19.31
Shackle	60	Heavy	10	1.65abc	25.05	1.16	48.56	3.99^f^	16.03
SEM2)				0.20	1.81	0.02	0.81	0.32	0.64
Main effects									
Cone			60	1.49^b^	21.14^b^	1.14b	46.75^b^	5.90^a^	19.44^a^
Shackle			60	1.89^a^	23.38^a^	1.21a	49.72^a^	5.32^b^	17.05^b^
SEM3)				0.08	0.74	0.01	0.33	0.13	0.26
	10		40	1.72	24.00	1.27^a^	50.15^a^	5.02^b^	17.45^b^
	30		40	1.73	21.18	1.15^b^	47.69^b^	5.64^a^	18.52^ab^
	60		40	1.63	21.62	1.12^b^	46.89^b^	6.17^a^	18.76^a^
	SEM4)			0.10	0.90	0.01	0.40	0.16	0.32
		Light	60	1.78	20.91^b^	1.18	49.73^a^	6.95^a^	20.30^a^
		Heavy	60	1.61	23.62^a^	1.18	46.75^b^	4.28^b^	16.18^b^
		SEM5)		0.08	0.74	0.01	0.33	0.13	0.26
ANOVA (p-value)									
R				**	*	***	***	**	***
D				0.73	0.06	***	***	***	*
W				0.15	*	0.89	***	***	***
R×D				**	0.23	*	**	***	0.50
R×W				0.22	0.75	***	0.07	0.19	*
D×W				0.09	0.15	**	*	0.14	0.09
R×D×W				0.16	0.45	0.18	0.62	**	0.56

SEM, standard error of the mean; ANOVA, analysis of variance.

1) Light (1.8±0.1 kg), heavy (2.8±0.1 kg).

2) Restraint method, restraint duration, and body weight effect (n = 10).

3) Restraint method effect (n = 60).

4) Restraint duration effect (n = 40).

5) Body weight effect (n = 60).

* p<0.05;

** p<0.01;

*** p<0.001.

a–d Means within a column-subgroup with no common superscripts are significantly different at p<0.05.
